# Cutaneous Diphtheria

**Published:** 1911-02-25

**Authors:** 


### Dermatology.

### CUTANEOUS DIPHTHERIA.

Even so long ago as the eighteenth century it was recognised that diphtheria may attack an open wound; few observers realise, however, that it is distinctly rare for cutaneous diphtheria to present any definite membrane, and it is on this account that the diagnosis is often missed. Bacteriological examinations in skin cases becoming more and more general, diphtheria bacilli are being found where they would formerly have been absolutely unsuspected. Several cases in point were brought before the Dermatological section of the British Medical Association by Dr. G. W. Dawson. Other observers have also recorded cases recently. The importance of arriving at the correct diagnosis is clearly great, for not only may the patient continue to have the skin affection for months if anti-diphtheritic serum treatment is not adopted, but also he is all this time an unsuspected source of dangerous infection to his family and to the community.

The commonest and, therefore, the most typical form of cutaneous diphtheria occurs in children, and has the appearance of an impetiginous eczema, nearly always affecting the head and face; often, but not always, associated with severe conjunctivitis, otorrhœa, or rhinitis; once seen and recognised the condition will seldom be overlooked again.

Seeing that the diphtheria bacillus may be simulated by certain common, but harmless, diphtheroid organisms, it is important to omit no cultural test in verifying its nature, and animal inoculation may be resorted to if need be. The malady is apt to be very resistant to ordinary treatment, lasting sometimes for *years* when its real nature is not recognised and the proper treatment adopted. Doubtless, some of the cases have not been primary diphtheria of the skin; various other affections may become secondarily inoculated with Klebs-Lœffler bacilli; but whether primary or secondary, the lesion invariably responds to antitoxin, and anti-diphtheritic serum is the correct treatment.

The following is a severe case recorded by Dr. Kenneth Wills in the Bristol *Medico-Chirurgical Journal*. The patient was a boy six years of age, who came under observation for enlarged glands in the neck on December 16. Six months previously he had had "an abscess which discharged through the right nostril." Since then glands had slowly increased in size on the left side of the neck without obviously affecting the general health. On December 16 a slight follicular inflammation of the left tonsil was noticed, but there was no pyrexia, and the patient was well enough to enjoy games and sing with the other children. The glands gradually increased during the next six weeks, and threatened to break down. The left tonsil, in spite of treatment by carbolic swabbing, potassium chlorate, and ferric chloride, slowly changed its appearance from a follicular tonsilitis to a punched-out ulcer. At the beginning of February the throat began to be painful, and tablets of menthaform were substituted for the carbolic swabbing. The skin over the shoulders became scaly in the middle of February, and this condition spread in a couple of weeks to the whole trunk and, to a lesser extent, to the thighs, arms, neck, and face, leaving the hands, feet, and scalp unaffected. By the end of the month, while the scaly condition was extending, the parts first involved were showing more inflammatory changes, and a small amount of serous discharge appeared, and the scales were shed, leaving a raw surface. At the end of the first week of March the whole of the trunk had become raw, the conjunctivæ were involved, the gums and lips were also ulcerated, and the whole condition of the child was pitiable. The discharge had rapidly become purulent and offensive, but no local treatment seemed in any way to allay the symptoms. The temperature did not run very high, but it was of the septic type.

Dr. Wills saw the case at this stage. The patient presented the appearance of a sufferer from profound toxæmia: his face was pallid; the lips were swollen, with sores all round them; there was a dressing over the glands behind the left ear on account of suppuration here; the boy was listless and apathetic, but would answer questions. Over the back, chest, and abdomen, with a few scattered lesions on the face and limbs, was a copious vesiculo-pustular rash. On the margins of the efflorescence the lesions were markedly discrete and of a conical shape, with a depressed centre, due to their being formed round the pilo-sebaceous follicles. Most of the lesions were pustular, though some were papular; whilst in the centre of the affected areas the old pustules had led to the formation of crusts and erosions. The intervening skin was in parts darkly erythematous, but round the more recent pustules there was only a narrow erythematous ring.

Inside the mouth there were one or two shallow ulcers on the lips and soft palate; one of these had a greyish pellicle. There was conjunctivitis of both eyes, the left being the worse, but the exudate was not characteristic of diphtheria. Cultures were made from the throat lesions, from the nose, from one eye, and from the skin lesions, and all were returned "positive Klebs-Lœffler." Anti-diphtheritic serum injections were started at once, and improvement set in both in the skin and in the eyes almost immediately.

All the pustules, back and front, disappeared, leaving only deeply pigmented discrete maculæ. The local treatment comprised boric ointment and zinc and mercury ointment applied on lint.

